# Changes in CSF sPDGFRβ level and their association with blood–brain barrier breakdown in Alzheimer’s disease with or without small cerebrovascular lesions

**DOI:** 10.1186/s13195-023-01199-5

**Published:** 2023-03-14

**Authors:** Xinyi Lv, Mengguo Zhang, Zhaozhao Cheng, Qiong Wang, Peng Wang, Qiang Xie, Ming Ni, Yong Shen, Qiqiang Tang, Feng Gao, China Aging Neurodegenerative Disorder Initiative (CANDI) Consortium

**Affiliations:** 1 Department of Neurology, Institute on Aging and Brain Disorders, The First Affiliated Hospital of USTC, Division of Life Sciences and Medicine, University of Science and Technology of China, Hefei, China; 2 Neurodegenerative Disorder Research Center, Division of Life Sciences and Medicine, University of Science and Technology of China, Hefei, China; 3 Department of Radiology, The First Affiliated Hospital of USTC, Division of Life Sciences and Medicine, University of Science and Technology of China, Hefei, China; 4 Department of Nuclear Medicine, The First Affiliated Hospital of USTC, Division of Life Sciences and Medicine, University of Science and Technology of China, Hefei, China; 5 Anhui Province Key Laboratory of Biomedical Aging Research, Hefei, China

**Keywords:** Alzheimer’s disease, Soluble platelet-derived growth factor receptor β, Amyloid-β, Cerebral small vessel disease, Blood–brain barrier

## Abstract

**Background:**

CSF-soluble platelet-derived growth factor receptor beta (sPDGFRβ) is closely associated with pericyte damage. However, the changes in CSF sPDGFRβ levels and their role in blood**–**brain barrier (BBB) leakage at different stages of Alzheimer’s disease (AD), with or without cerebral small vessel disease (CSVD) burden, remain unclear.

**Methods:**

A total of 158 individuals from the China Aging and Neurodegenerative Disorder Initiative cohort were selected, including 27, 48, and 83 individuals with a clinical dementia rating (CDR) score of 0, 0.5, and 1**–**2, respectively. CSF total tau, phosphorylated tau181 (p-tau181), Aβ40, and Aβ42 were measured using the Simoa assay. Albumin and CSF sPDGFRβ were measured by commercial assay kits. CSVD burden was assessed by magnetic resonance imaging.

**Results:**

CSF sPDGFRβ was the highest level in the CDR 0.5 group. CSF sPDGFRβ was significantly correlated with the CSF/serum albumin ratio (Q-alb) in the CDR 0–0.5 group (*β* = 0.314, *p* = 0.008) but not in the CDR 1–2 group (*β* = − 0.117, *p* = 0.317). In the CDR 0–0.5 group, CSF sPDGFRβ exhibited a significant mediating effect between Aβ42/Aβ40 levels and Q-alb (*p* = 0.038). Q-alb, rather than CSF sPDGFRβ, showed a significant difference between individuals with or without CSVD burden. Furthermore, in the CDR 0.5 group, CSF sPDGFRβ was higher in subjects with progressive mild cognitive impairment than in those with stable mild cognitive impairment subjects (*p* < 0.001). Meanwhile, CSF sPDGFRβ was significantly associated with yearly changes in MMSE scores in the CDR 0.5 group (*β* = − 0.400, *p* = 0.020) and CDR 0.5 (A+) subgroup (*β* = − 0.542, *p* = 0.019).

**Conclusions:**

We provide evidence that increased CSF sPDGFRβ is associated with BBB leakage in the early cognitive impairment stage of AD, which may contribute to cognitive impairment in AD progression.

**Supplementary Information:**

The online version contains supplementary material available at 10.1186/s13195-023-01199-5.

## Background

Alzheimer’s disease (AD) is a major cause of dementia in elderly populations. There are numerous risk factors leading to AD occurrence and development, such as age, depression, obesity, diabetes, and hypertension [1–4]. Recently, in addition to two feature pathologic characterizations, amyloid-β (Aβ) plaques and hyperphosphorylation tangles, cerebrovascular dysfunction has also been recognized to contribute to AD [5]. The blood–brain barrier (BBB) is a physical barrier between the peripheral circulation and the central nervous system (CNS), and its breakdown contributes to the neurodegenerative process in many neurologic diseases [5–8]. In AD, early BBB breakdown is known to occur before cognitive impairment [9, 10]. Meanwhile, several studies have indicated that Aβ accumulation is generally accompanied by the occurrence of cerebral small vessel damage in patients with AD, indicating the possible synergistic effect of cerebrovascular damage [11–13].

Platelet-derived growth factor receptor beta (PDGFRβ) is a type of tyrosine kinase receptor expressed by pericytes and vascular smooth muscle cells (SMCs) and usually serves as a biomarker of pericytes as PDGFRβ expression in pericytes is much higher than that in SMCs [5, 14]. PDGFRβ is essential for the proliferation, migration, and recruitment of pericytes and SMCs. It can be cleaved by proteases such as metallopeptidase domain 10 in pericytes but not in SMCs. Soluble PDGFRβ (sPDGFRβ) in the cerebrospinal fluid (CSF) has been suggested to be closely associated with pericyte and BBB damage [5, 9]. A mouse model study demonstrated that pericyte injury resulted in an elevated sPDGFRβ concentration in CSF [9]. Moreover, in patients with mild cognitive impairment (MCI), CSF sPDGFRβ levels increased and were related to increased BBB permeability in the hippocampus, CA1 region, and dentate gyrus [5]. In patients with AD, sPDGFRβ levels in CSF were noticeably higher than in subjects with normal cognition (CN) and were positively correlated with CSF albumin, t-tau, and p-tau181 levels [15]. Meanwhile, another study revealed a significant increase in CSF sPDGFRβ levels only in Aβ and p-tau181-positive patients with AD [16].

The China Aging and Neurodegenerative Disorder Initiative (CANDI) cohort recruited elderly participants with or without cognitive impairment for early diagnosis and prediction of AD in the Chinese Han population [17]. In this study, we selected 158 participants, including individuals with normal cognition (CDR 0) and patients with MCI (CDR 0.5) or AD (CDR 1–2), from the CANDI cohort. We investigated the CSF sPDGFRβ levels and analyzed the association of CSF sPDGFRβ with BBB breakdown at different stages of cognitive impairment. We then evaluated the associations between CSF sPDGFRβ levels and CSF Aβ42, Aβ40, p-tau, and t-tau. As cerebrovascular damage is another pivotal factor that contributes to BBB leakage [18], we compared the difference in CSF sPDGFRβ levels among the groups with or without cerebral small vessel disease (CSVD) burden. Moreover, to investigate the potential effect of pericyte damage on AD progression, we compared CSF sPDGFRβ levels in the CDR 0.5 group showing different cognitive decline rates.

## Methods

### Participants

The CANDI study was launched in 2018; it was a longitudinal study including individuals with normal cognition (CN), mild cognitive impairment (MCI), and dementia. All participants were recruited from the First Affiliated Hospital of the University of Science and Technology of China (USTC) in November 2018. CSF samples, plasma samples, imaging data, and cognition measurements were available and were used for this study [17].

For our study, 158 participants were from the CANDI cohort, and complete clinical data were required, after a detailed cognitive assessment, including the Mini-Mental State Examination (MMSE) and clinical dementia rating (CDR) scores (Fig. S1). Twenty-seven participants (MMSE ≧ 24), with conditions such as primary headache, facial neuritis, dizziness, and ophthalmoplegia, with normal CSF cell numbers and protein to exclude intracranial hemorrhage, infections and inflammation of the central nervous system, and significant blood–brain barrier damage, were included in the CDR 0 group. A total of 131 patients with cognitive impairment (CI) were divided into two groups: the CDR 0.5 group (48 cases) and the CDR 1–2 group (83 cases) based on the NIA-AA criteria (2011) and CDR score [19]. These criteria excluded vascular dementia (defined by a history of a stroke temporally related to the onset or worsening of cognitive impairment or the presence of multiple or extensive infarcts or severe white matter hyperintensity burden), Lewy body dementia, behavioral variant frontotemporal dementia, Parkinson’s disease dementia, semantic variant primary progressive aphasia, non-fluent/agrammatic variant primary progressive aphasia or other active neurological diseases, or a non-neurological medical comorbidity that could have a substantial effect on cognition. Meanwhile, to further investigate the association of CSF sPDGFRβ with Aβ pathology, we reclassified the whole cohort, and cases that were positive based on the ^18^F-florbetapir PET and/or CSF Aβ42/40 ratio were included in the A+ group. All cases in the CDR 1–2 group were A+, and only 3 cases were A+ in the CDR 0 group, but in the CDR 0.5 group, there were 20 A− cases and 28 A+ cases, using accepted cutoff values (0.06423) and/or visual judgment for ^18^F-florbetapir PET in the CANDI study [17, 20]. *APOE* genotypes were determined as described previously [21]. Each patient in this study provided written informed consent in accordance with the Declaration of Helsinki. The protocols used in this study were reviewed and approved by the ethics committee of our hospital.

### CSF collection and measurement for AD markers

CSF specimens from patients or controls in the above three groups were collected using lumbar puncture (LP) in the morning after an overnight fast. After confirming a clear appearance of the CSF sample, it was aliquoted in polypropylene tubes and stored at − 80 °C until measurements were taken. The CSF samples were vortexed and centrifuged at 10000×*g* for 5 min before dilution. CSF Aβ40, Aβ42, P-tau181, and total tau were measured by the Simoa kits (Quanterix, 103714, 101195). CSF sPDGFRβ (EHPDGFRB, Thermo Fisher Scientific) and albumin levels were measured by the corresponding commercial assay kits (EHALB, Thermo Fisher Scientific).

### Analysis of the CSF/plasma albumin ratio (Q-alb)

Noanticoagulative blood samples were collected by venipuncture during the collection of CSF specimens. The collected blood samples were centrifuged, and serum albumin concentrations were determined using a bromocresol green dye binding assay (ADVIA 1800; Siemens, Berlin, Germany). The Q-alb value was calculated using the following formula: (CSF albumin/serum albumin) × 1000.

### Determination of the total MRI burden of CSVD

All images were acquired by using GE DISCOVER 750w 3.0T MRI scanner (GE Healthcare, USA), including DWI/FLAIR/SWI and T2-weighted images, which were assessed by two experienced neuroradiologists blinded to clinical information. According to the recently described score for small vascular lesions [22], we rated the total MRI burden of CSVD on an ordinal scale from 0–4 by counting the presence of each of the four MRI features of CSVD: lacunes (1 point if ≥ 1 lacune present), any cerebral microbleed (1 point if present), moderate to severe perivascular spaces (grade 2–4) in the basal ganglia (1 point if present), and periventricular white matter hyperintensities (WMH) meeting or exceeding Fazekas scale 3 and/or deep WMH meeting or exceeding Fazekas scale 2–3 (1 point if present). Based on the results of the analysis, the patients were divided into three groups: CSVD burden scores of 0, 1, and 2–4.

### Statistical analyses

The IBM SPSS 23.0 software for Windows (SPSS; Chicago, IL, USA), GraphPad Prism 7.0 (GraphPad Software; La Jolla, CA, USA), and R 4.0.4 (ggplot2, ggpubr, mediation, and QuantPsyc) were used for the analysis. Statistical significance in all two-sided tests was defined as *p* value < 0.05. For describing demographic data, the Wilcoxon signed-rank test was used for continuous variable intergroup comparisons, and chi-square analysis was used for categorical variables. AD biofluid biomarker measurements including Aβ42, Aβ40, p-tau, t-tau, and the Aβ42/Aβ40 ratio were compared using analysis of covariance (ANCOVA), with age, sex, and the *APOE* genotype as covariates. ANCOVA was also used to compare CSF sPDGFRβ and Q-alb levels between different cognition groups. A multiple linear regression model was adopted to evaluate the associations between CSF sPDGFRβ, Q-alb, and AD biomarkers. In multiple linear regression models, ratios of Q-alb much greater or lower than the triple standard deviation from the mean value were regarded as outliers and discarded. The Q-albumin ratio and concentration measures of CSF sPDGFRβ were ln-transformed when necessary, and the Shapiro–Wilk test was used to test the normality of the transformed data. Age, sex, and *APOE* genotype were included in multiple linear regression models as covariances to adjust the effects. The extent of pericyte injury contributing to BBB damage was determined using mediation analysis. This was based on a multiple linear regression model adjusted for age, sex, and *APOE* genotype. Ranked data, such as CSVD scores, are shown as the median of the interquartile range (IQR).

## Results

### Demographics

As shown in Table 1, 158 patients were selected, including individuals in the CDR 0 group (27 cases), CDR 0.5 group (48 cases), and CDR 1–2 group (83 cases). In the CDR 1–2 group, the proportion of female patients was higher, as predicted (68.67%). The mean age of the CDR 0 group was significantly lower than that of the CDR 1–2 group (*p* = 0.013). MMSE scores were significantly different among the three groups. As expected, there were more *APOE e4* gene carriers in the CDR 0.5 group (45.83%) and CDR 1–2 group (63.86%) than in the CDR 0 (14.81%) group.

**Table 1:** Demographic characteristics of subjects

		CDR = 0	CDR = 0.5	CDR 1~2
**No.**		27	48	83
**Male, %**		15/27 (55.56%)^c^	19/48 (39.58%)	26/83 (31.33%)^a^
**Age, years**	Mean ± SD	60.70 ± 6.64^c^	63.19 ± 8.96	64.69 ± 7.57^a^
	Median	61.00	64.00	67.00
	95% CI	58.08, 63.33	60.59, 65.79	63.03, 66.34
**Education, years**	Mean ± SD	8.30 ± 3.91	9.29 ± 4.78^c^	6.93 ± 4.70^b^
	Median	9.00	10.00	8.00
	95% CI	6.75, 9.84	7.90, 10.68	5.90, 7.95
**MMSE, scores**	Mean ± SD	27.51 ± 2.34^b, c^	22.60 ± 4.16^a, c^	12.18 ± 5.86^a, b^
	Median	28.00	23.50	13.00
	95% CI	26.59, 28.45	21.40, 23.81	11.35, 13.91
***APOE e4*** **carriers,** ***n*** **(%)**		4 (14.81%)^b, c^	22 (45.83%)^a, c^	53 (63.86%)^a, b^
**Amyloid positive, %**		3 (11.11%)	28 (58.33%)	83 (100.00%)
**Q-alb (× 10**^**3**^**)**	Mean ± SD	7.10 ± 2.97^c^	8.57 ± 3.47	9.40 ± 4.06^a^
	Median	6.51	8.10	8.63
	95% CI	5.93, 8.28	7.52, 9.61	8.48, 10.32
**CSF sPDGFRβ**	Mean ± SD	179.83 ± 51.23^b^	252.50 ± 57.11^a, c^	219.15 ± 61.05^b^
	Median	166.66	249.79	220.57
	95% CI	0.06, 0.07	235.34, 269.65	205.65, 232.65
**CSF p-tau, pg/ml**	Mean ± SD	33.84 ± 8.81^b, c^	76.67 ± 55.72^a, c^	130.87 ± 86.29^a, b^
	Median	34.90	56.00	110.00
	95% CI	30.12, 37.55	60.31, 93.03	112.03, 149.71
**CSF t-tau, pg/ml**	Mean ± SD	74.44 ± 21.91^b, c^	124.73 ± 66.56^a, c^	198.13 ± 137.29^a, b^
	Median	75.53	108.00	164.80
	95% CI	65.59, 83.29	105.18, 144.27	168.16, 228.11
**CSF Aβ42, pg/ml**	Mean ± SD	700.61 ± 338.99^b, c^	463.52 ± 286.36^a, c^	290.24 ± 153.02^a, b^
	Median	669.60	368.95	273.20
	95% CI	560.68, 840.54	379.44, 547.60	256.61, 323.86
**CSF Aβ40, pg/ml**	Mean ± SD	7428.22 ± 3220.53	6972.30 ± 2511.04	6288.86 ± 2897.36
	Median	8000	6712	6112
	95% CI	6098.86, 8757.59	6226.62, 7717.99	5639.89, 6937.84
**CSF Aβ42/Aβ40 ratio**	Mean ± SD	0.094 ± 0.02^b, c^	0.064 ± 0.03^a, c^	0.046 ± 0.01^a, b^
	Median	0.10	0.06	0.04
	95% CI	0.09, 0.10	0.06, 0.07	0.04, 0.05

*p-tau* phosphorylated tau, *t-tau* total tau

*p* < 0.05 was considered statistically significant

^a^Significant values vs CDR 0

^b^Significant values vs CDR 0.5

^c^Significant values vs CDR 1–2

### Comparisons and association between CSF sPDGFRβ and Q-alb

**Fig. 1:**
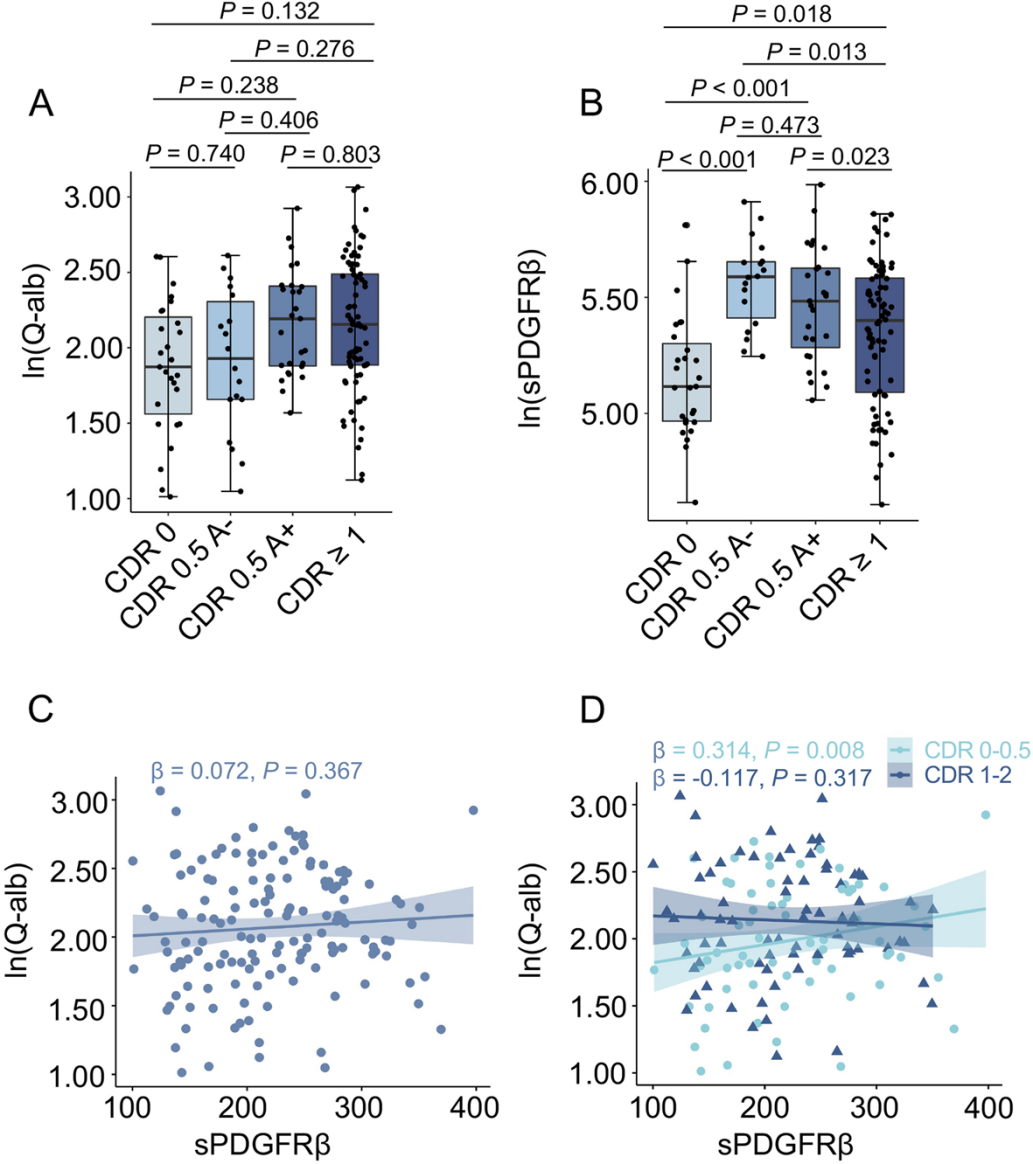
Comparisons and correlation analysis of CSF sPDGFRβ levels as well as Q-alb levels in groups with different cognitive stages. Analysis of covariance (ANCOVA) was used to perform multiple comparisons of ln-transformed s Q-alb ratios (**A**) and ln-transformed CSF sPDGFRβ concentrations (**B**) between CDR 0 group, CDR 0.5 A−, CDR 0.5 A+ group, and CDR 1–2 group. Sex, age, and *APOE* genotype were adjusted as covariates. The middle line of the boxplot represents the median value of the group, and the lower and upper lines represent the first and third quantiles, respectively. Multiple linear regression models were used to analyze the correlation between ln-transformed Q-alb ratios and CSF sPDGFRβ levels in the whole cohort (**C**) and in different cognitive groups (**D**). Sex, age, and *APOE* genotype were adjusted as covariates. Standardized regression coefficients and *p* values are presented. A value of *p* < 0.05 was considered statistically significant after false discovery rate post hoc analysis for multiple comparisons

The Q-alb exhibited no significant difference among the CDR 0, CDR 0.5 A−, CDR 0.5 A+, and CDR 1–2 groups (Fig. 1A). Interestingly, CSF sPDGFRβ in the CDR 0.5 A− and CDR 0.5 A+ groups was highly significantly elevated compared with that in CDR 0 patients (Fig. 1B, *p <* 0.001). However, its level was reduced in the CDR 1–2 group compared to that in the CDR 0.5 A− group (*p =* 0.013) and CDR 0.5 A+ group (*p* = 0.023). In addition, we compared the CSF sPDGFRβ levels between subjects with a different *APOE e4* gene carrier status or different sex. In the CDR 0, CDR 0.5, or CDR 1–2 groups, no significant difference was observed between *APOE e4* gene carriers and non-carriers or males and females (Fig. S2A and Fig. S2B). Meanwhile, CSF sPDGFRβ levels correlated with age (*p* = 0.040, Fig. S2C). We calculated the contributions of age, sex, and *APOE e4* genotype to the variance in CSF sPDGFRβ levels. The proportions of explained variances in age, sex, and *APOE* genotype were 3.10%, 3.21%, and 0.14%, respectively, for CSF sPDGFRβ (Fig. S2D). Moreover, the correlation between Q-alb and sPDGFRβ levels was also assessed. As shown in Fig. 1C, D, there was no correlation between Q-alb and sPDGFRβ in all participants or in the CDR 1–2 group. However, a strong positive correlation between Q-alb and sPDGFRβ was observed in the CDR 0–0.5 group (*β* = 0.314, *p* = 0.008).

### Evaluations of the association between CSF sPDGFRβ and AD core biomarkers

We then performed multiple linear analyses to evaluate the association between CSF sPDGFRβ and t-tau, p-tau, Aβ40, Aβ42, and the Aβ42/Aβ40 ratio. For patients with a CDR of 0–0.5, significant correlations were observed between CSF sPDGFRβ and p-tau (*β* = 0.302, *p* = 0.014), t-tau (*β* = 0.350, *p* = 0.003), and Aβ42/Aβ40 ratio (*β* = − 0.249, *p* = 0.049). In patients with CDR 1–2, there were positive correlations between sPDGFRβ and p-tau (*β* = 0.342, *p* = 0.003), t-tau (*β* = 0.374, *p* < 0.001), Aβ40 (*β* = 0.330, *p* = 0.004), and Aβ42 (*β* = 0.246, *p* = 0.027). Q-alb was correlated with t-tau (*β* = 0.266, *p* = 0.024) in the CDR 0–0.5 group, whereas a significant correlation was observed between Q-alb and Aβ42/Aβ40 (*β* = 0.294, *p* = 0.013) in the CDR 1–2 group (Table 2).

**Table 2:** Characteristics of multiple regression models between CSF sPDGFRβ, Q-alb, and AD biomarkers

			t-tau	p-tau	Aβ40	Aβ42	Aβ42/40
**CSF sPDGFR**	**CDR 0~2**	*β*	0.252	0.213	0.240	0.137	− 0.072
		*p* value	**0.002**	**0.011**	**0.003**	0.102	0.426
	**CDR 0~0.5**	*β*	0.350	0.302	0.132	0.011	− 0.249
		*p* value	**0.003**	**0.014**	0.276	0.931	**0.049**
	**CDR 1~2**	*β*	0.374	0.342	0.330	0.246	− 0.071
		*p* value	**< 0.001**	**0.003**	**0.004**	**0.027**	0.550
**Q-alb**	**CDR 0~2**	*β*	0.175	0.117	0.034	− 0.053	− 0.106
		*p* value	**0.032**	0.154	0.680	0.521	0.228
	**CDR 0~0.5**	*β*	0.266	0.223	− 0.029	− 0.101	− 0.244
		*p* value	**0.024**	0.065	0.807	0.407	0.052
	**CDR 1~2**	*β*	0.089	− 0.019	0.118	0.187	0.294
		*p* value	0.447	0.870	0.327	0.099	**0.013**

*p* < 0.05 was considered statistically significant

### CSF sPDGFRβ-mediated effects of Aβ pathology on BBB permeability

**Fig. 2:**
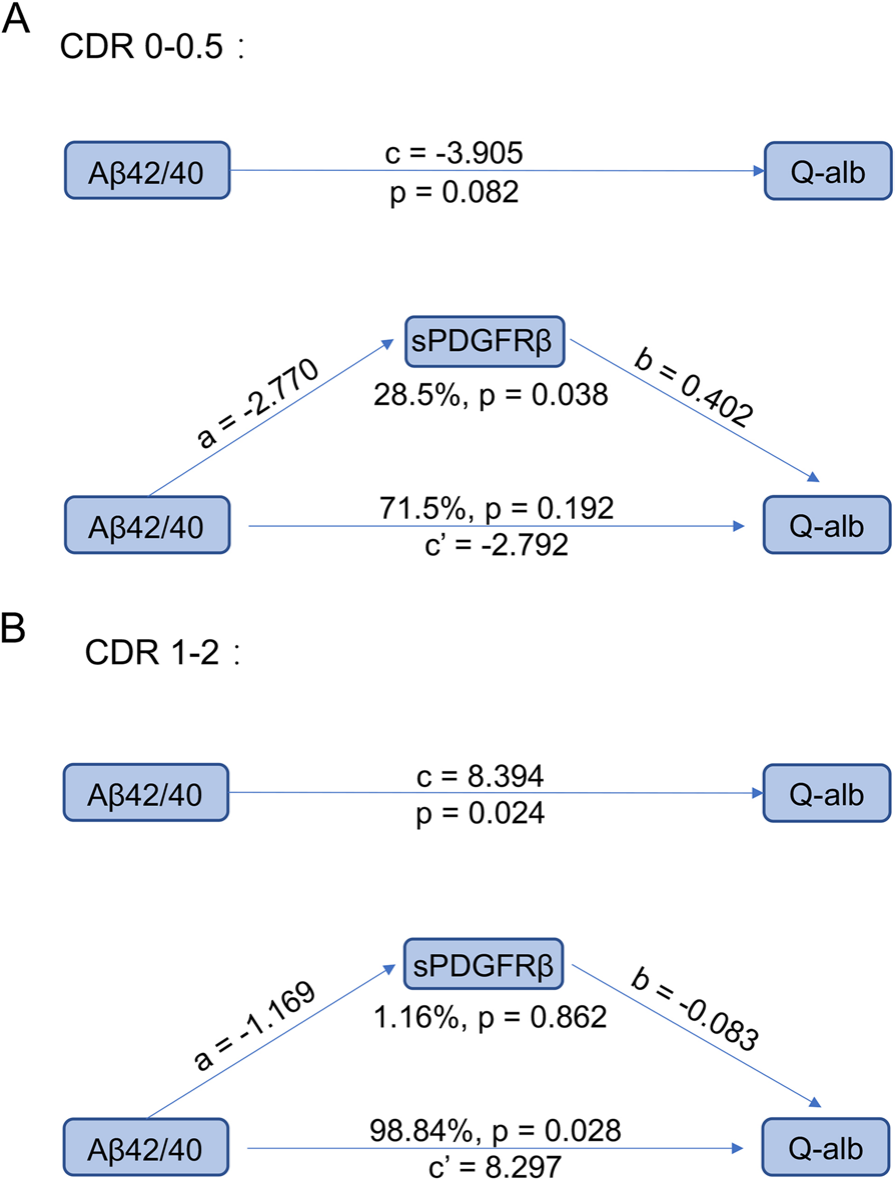
Mediation analysis regarding the proportion to which CSF sPDGFRβ alteration affects the process of BBB damage. Mediation analysis included the following variables: the concentration of CSF sPDGFRβ was treated as a mediator, Q-alb was set as the dependent variable, and Aβ42/Aβ40 ratio was set as the independent variable. Analyses based on multiple linear regression models with sex, age, and *APOE* genotype was adjusted as covariates. The values for CSF sPDGFRβ levels and the Q-alb ratio were ln-transformed. A value of *p* < 0.05 was considered statistically significant

BBB breakdown and pericyte injury have been suggested to occur before cognitive impairment in patients [5]. Meanwhile, it has been suggested that Aβ pathology is suggested to be associated with BBB damage [15, 23]. To investigate whether the association between Aβ pathology and BBB damage was regulated by CSF sPDGFRβ, we performed a mediation analysis [24]. As shown in Fig. 2, mediation analysis revealed that from the cognitively unimpaired period to the early stage of cognitive impairment (CDR 0–0.5), CSF sPDGFRβ-mediated effect accounted for a statistically significant proportion of Aβ toxic effects on the BBB (*p* = 0.038) (28.5% for Aβ42/Aβ40). Meanwhile, there was no significant direct effect (i.e., ADE) or CSF sPDGFRβ-mediated effects (i.e., ACME) of Aβ42 or Aβ40 on Q-alb ratios. However, in the CDR 1–2 group, Aβ42/Aβ40 changed its CSF sPDGFRβ-mediated effect on BBB damage (1.16%, *p* = 0.862) to a direct effect on BBB damage (98.84%, *p* = 0.028).

### Associations between the Q-alb ratio or CSF sPDGFRβ level and CSVD burden

**Fig. 3:**
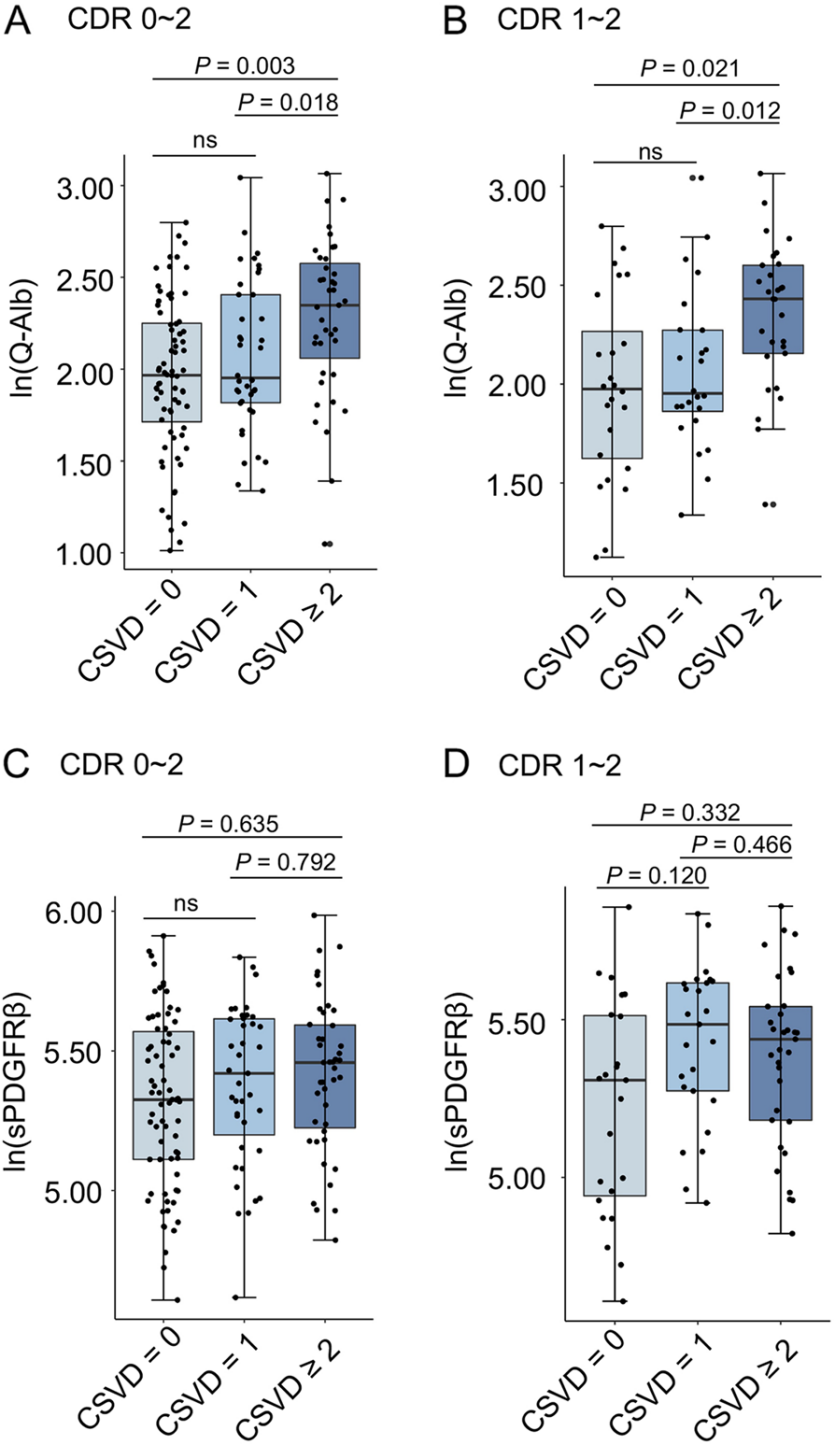
Comparisons of Q-alb ratios and CSF sPDGFRβ levels among the groups with various CSVD burdens. The box-and-whisker plot presents the multiple comparisons of ln-transformed Q-alb ratios among the groups with different CSVD burdens in the whole cohort (**A**) and in the CDR 1–2 subjects (**B**), as well as the ln-transformed CSF sPDGFRβ levels among groups divided by different CSVD scores in the whole cohort (**C**) and in the CDR 1–2 subjects (**D**). A value of *p* < 0.05 was considered statistically significant after Bonferroni post hoc analysis for multiple comparisons

As BBB breakdown is closely associated with cerebral vascular damage, it is necessary to analyze whether the association between CSF sPDGFRβ and Q-alb varies in subjects with or without CSVD burden. The characteristics of CSVD burden in all participants were evaluated, which confirmed significant differences in the combined total CSVD burden (*p* < 0.001), PVWMH (*p* < 0.001), DWMH (*p* = 0.035), and cerebral microbleeds (*p* = 0.028) among the CDR 0, 0.5, and 1–2 groups (Table 3). The levels of Q-alb and CSF sPDGFRβ at each CDR stage with different CSVD burdens were compared. Q-alb was significantly increased only in individuals with higher CSVD burdens (scores of 2 and higher) (Fig. 3A and B). However, CSF sPDGFRβ levels were not significantly different among the three groups (Fig. 3C, D).

**Table 3:** Characteristics of the patients with CSVD in CDR 0, 0.5, and 1–2

	Score	CDR 0 (*N* = 27)	CDR 0.5 (*N* = 48)	CDR 1–2 (*N* = 83)	*p* value
CSVD, *n* (%)	0	20 (74.07%)	30 (62.50%)	25 (30.12%)	< 0.001
	1	6 (22.22%)	8 (16.67%)	25 (30.12%)	
	≥ 2	1 (3.70%)	10 (20.83%)	33 (39.76%)	
PVWMH, *n* (%)	0	22 (81.48%)	24 (50.00%)	26 (31.33%)	< 0.001
	1	5 (18.52%)	17 (35.42%)	29 (34.94%)	
	2	0 (0.00%)	6 (12.50%)	22 (26.51%)	
	3	0 (0.00%)	1 (2.08%)	6 (7.23%)	
DWMH, *n* (%)	0	23 (85.19%)	30 (62.50%)	42 (50.60%)	0.035
	1	4 (14.81%)	17 (35.42%)	32 (38.55%)	
	2	0 (0.00%)	1 (2.08%)	6 (7.23%)	
	3	0 (0.00%)	0 (0.00%)	3 (3.61%)	
CMBn, median (IQR)		0 ± 0	0 ± 0	0 ± 4	0.028

*p* < 0.05 was considered statistically significant.

*PVWMHs* periventricular spaces white matter hyperintensity Fazekas score, *DWMHs* deep spaces white matter hyperintensity Fazekas score, *CMBn* cerebral microbleeds numbers

### Baseline CSF sPDGFRβ is associated with worsening cognitive function in the future

**Fig. 4:**
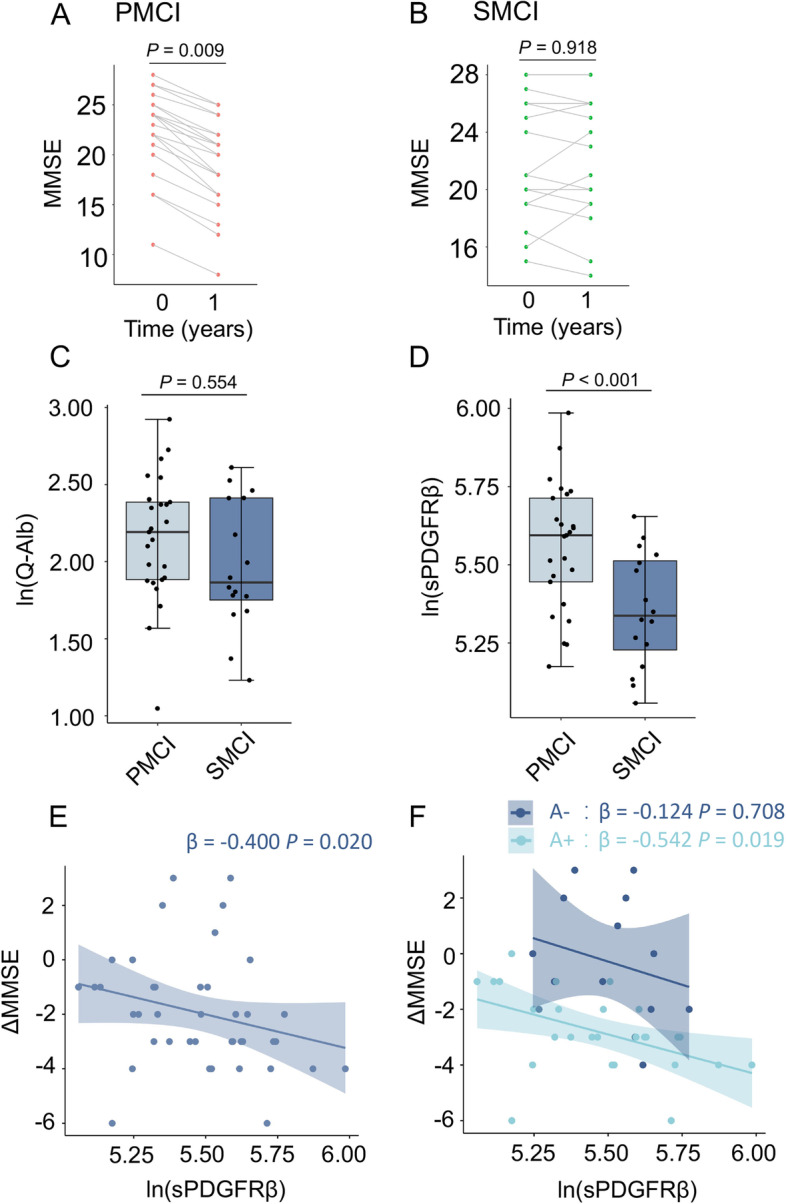
Comparison of baseline levels of Q-alb and CSF sPDGFRβ levels between two individuals with differential development in the CDR 0.5 group. Paired scatter plots show the longitudinal trajectories of cognitive changes in subjects with PMCI (**A**) and SMCI (**B**). Boxplots show the multiple comparisons of ln-transformed Q-alb ratios (**C**) and ln-transformed CSF sPDGFRβ concentrations (**D**) between different groups clarified by cognition deterioration rate, adjusted by age, sex, *APOE* genotype, and CSF Aβ42/40 ratio. The regression plots show an association between baseline CSF sPDGFRβ and cognitive decline in the CDR 0.5 group (**E**) and in the CDR 0.5 group A+ subgroup (**F**). Age, sex, and *APOE* genotype were adjusted. A value of *p* < 0.05 was considered statistically significant

A 1-year longitudinal follow-up study was conducted in the CDR 0.5 group. Among these, 25 gradually progressing cases were defined as the progressive mild cognitive impairment (PMCI) group, and one patient met the diagnostic criteria for AD (Fig. 4A, *p* = 0.009). The remaining 19 patients with stable disease pathology were defined as the stable mild cognitive impairment (SMCI) group (Fig. 4B, *p* = 0.918). No significant difference was observed between the PMCI and SMCI groups regarding the baseline age, sex ratio, years of education, MMSE, *APOE* genotype, Aβ40, and Aβ42 levels (Table 4). However, the concentrations of t-tau (*p* = 0.004) and p-tau (*p* = 0.004) were significantly higher in the PMCI group, whereas the ratio of Aβ42/Aβ40 was lower in the PMCI group (*p* = 0.005) (Table 4). Q-alb showed no distinctive difference between the PMCI and SMCI groups (*p* =0.554, Fig. 4C). However, CSF sPDGFRβ was significantly higher in the PMCI group than in the SMCI group (*p* < 0.001, Fig. 4D), even when adjusted for Aβ42/40. Baseline CSF sPDGFRβ levels were correlated with MMSE yearly change in the CDR 0.5 group (*β* = − 0.400, *p* = 0.020, Fig. 4E) and CDR 0.5 A+ group (*β* = − 0.542, *p* = 0.019), while the correlation in the A− group was not statistically significant (Fig. 4F).

**Table 4:** The characteristics of the patients in PMCI and SMCI

	PMCI (*N* = 25)	SMCI (*N* = 19)	*p* value
Age, years	65.04 ± 8.66	62.84 ± 8.36	0.302
Male, *n* (%)	13 (52%)	5 (26.32%)	0.138
Edu, years	10.12 ± 4.43	7.68 ± 5.18	0.084
MMSE, scores	22.28 ± 4.02	22.05 ± 4.27	0.721
CSF t-tau, pg/ml	152.97 ± 78.86	97.60 ± 32.74	0.004
CSF p-tau, pg/ml	103.64 ± 62.79	52.36 ± 28.39	0.004
CSF Aβ40, pg/ml	6822.7 ± 2590.69	6993.4 ± 2496.10	0.602
CSF Aβ42, pg/ml	362.7 ± 203.10	540.2 ± 345.81	0.064
CSF Aβ42/40 ratio	0.054 ± 0.02	0.07 ± 0.03	0.005

*p* < 0.05 was considered statistically significant

## Discussion

In this study, we investigated the changes in CSF sPDGFRβ levels in patients with different stages of cognitive impairment (CDR 0–2), including MCI or AD, with or without CSVD burden. Some interesting findings were observed: (1) the most significant increase in CSF sPDGFRβ levels was found in CDR 0.5 group; (2) in the CDR 0–0.5 group, CSF sPDGFRβ levels were significantly associated with Q-alb and partially mediated Aβ pathology-induced Q-alb change; (3) in the CDR 1–2 group, CSF sPDGFRβ was associated with AD core biomarkers, and no significant mediating effect was observed in the relationship between Aβ pathology and Q-alb; (4) CSF sPDGFRβ levels were not different between the groups with CSVD burden; and (5) in the CDR 0.5 group, the increased CSF sPDGFRβ levels were associated with accelerated cognitive decline.

Elevation of CSF sPDGFRβ levels in humans and animal models indicates pericyte injury and is linked to BBB breakdown [5, 9, 15, 25–27]. Pericytes are a crucial unit of the neurovascular system and are reported to be closely associated with BBB integrity [15, 28]. More importantly, pericyte injury or degeneration plays a key role in the occurrence of AD [25, 29]. Previous reports have confirmed that pericyte injury caused by Aβ contributes to BBB breakdown [25]. Meanwhile, CSF sPDGFRβ was found to be correlated with DCE-MRI measures of BBB permeability in the early stage of cognitive dysfunction [5]. In this study, we only observed the association of CSF sPDGFRβ and Q-alb in the CDR 0–0.5 group but not in the CDR 1–2 group. Thus, it can be considered that the accumulation of toxic Aβ and other risk factors causes damage to pericytes, thereby weakening the integrity of the BBB in the early stage of cognitive impairment. However, as the disease progresses, many AD-associated pathological mechanisms, such as the inflammatory response, astroglial dysfunction, and neuronal injury, further result in physical damage to other components in the neurovascular unit (NVU) and functional loss in the BBB [30]. Interestingly, the mediation analysis model also revealed that Aβ-mediated pericyte damage plays a major role in BBB damage at the early cognitive damage stage but not in the dementia stage of AD. Thus, the accumulated toxic Aβ-mediated BBB destabilization occurs by inducing endothelial cell and vascular astrocyte dysfunction [31, 32], as well as through pericyte damage.

In the previous studies, Nation et al. [5] mainly focused on the changes in CSF sPDGFRβ in individuals with early cognitive impairment. They found that sPDGFRβ levels in CSF were higher in the CDR 1 group than in the CDR 0.5 group. However, Sweeney et al. [33] reported that there was no difference in CSF sPDGFRβ between the CDR 1 group and the CDR 0.5 group. In these studies, CSF sPDGFRβ levels in either the CDR 0.5 or CDR 1 group were consistently higher than those in the CDR 0 group, which was also observed in the present study. However, we observed that the CSF sPDGFRβ level was highest in the CDR 0.5 group. In contrast to previous studies that included subjects in the CDR 0.5 and CDR 1 groups by clinical diagnosis, the subjects in the CDR 1–2 group were biologically diagnosed with AD in the present study. The inconsistent results may be ascribed to the different diagnostic criteria in these studies. Meanwhile, the age of the subjects in the present study was lower than that in the two other studies, which may also contribute to the inconsistent results.

Although elevated CSF sPDGFRβ levels in AD have been verified in several independent studies [5, 9, 15, 21, 23], decreased CSF sPDGFRβ levels in patients with CDR 1–2 subjects compared to that in patients with CDR 0.5 is intriguing. A possible reason for this is that the total number of pericytes decreases in the middle and late stages of AD, which may cause a reduction in sPDGFRβ concentration. A decrease in the total number of pericytes in the hippocampus of APP/PS1 and 5XFAD mice has also been reported [34, 35]. An increased loss of pericytes was also identified in the hippocampus and retina of patients with AD [35, 36]. Pericyte loss has also been associated with increased Aβ40 and Aβ42 burden in the retinal vasculature [35]. Thus, CSF sPDGFRβ could be regarded as a biomarker for BBB damage in the early stage of AD, but not in the whole AD continuum.

We also explored the possible relationship between pericyte damage and cerebral small vessel lesions in AD progression. CSF Q-alb was found to be significantly increased in individuals with CSVD burden ≥ 2, whereas CSF sPDGFRβ levels were similar among the groups with or without CSVD burden. Previous studies have suggested that cerebral small vessel lesions could cause cognitive impairment independent of Aβ [18]. The prevalence of vascular risk factors (VRFs) is also a common measure to assess vascular load in patients with AD. Nation et al. reported that increased CSF sPDGFRβ levels in individuals with vascular damage and BBB dysfunction were not associated with VRFs [5]. Thus, these data suggest that CSF sPDGFRβ is more related to AD pathology-mediated pericyte damage.

Numerous studies have indicated that BBB breakdown is a marker of cognitive dysfunction [5, 24], and our longitudinal study results indicate that baseline CSF sPDGFRβ levels are associated with accelerated cognitive decline in the CDR 0.5 group and in the CDR 0.5 (A+) subgroup. Moreover, CSF sPDGFRβ levels in individuals with worsening cognition are much higher than those in individuals with stable cognition. Increased sPDGFRβ levels reflect more serious pericyte damage, and pericytes are of vital importance to BBB integrity and neurovascular unit function. As accelerated BBB breakdown and cerebral blood flow reduction were observed in pericyte-deficient mice [37], and pericyte loss influenced AD-like neurodegeneration in the *APP*^*sw/0*^ mouse model [25], it is reasonable to infer that in patients with MCI, higher CSF sPDGFRβ levels suggest more serious BBB damage, which may accelerate AD progression. Furthermore, pericyte injury initiates a reduction in Aβ clearance [25, 38, 39], which may further lead to Aβ deposition and accelerate the progression of AD pathology [40, 41].

Our study had some limitations. (1) The sample size was relatively small and was based on a single-center clinical cohort, with participants mainly recruited from eastern China. To obtain more accurate and general results, a larger population-based multicenter clinical study is needed. (2) Longer observation times and more follow-up cases are necessary to improve the statistical effectiveness of such longitudinal studies. (3) Other important biomarkers for BBB leakage, such as DCE-MRI data, were unavailable. In the future, a more comprehensive study should improve our understanding of the role of pericyte damage and BBB breakdown in AD progression.

In conclusion, our study characterized the changes in CSF sPDGFRβ levels in different cognitive stages of AD and analyzed the relationship between CSF sPDGFRβ and AD core biomarkers, CSVD burden, and BBB breakdown. These results suggest that the contribution of pericyte injury to BBB damage varies during the progression of AD. The association between CSF sPDGFRβ levels and cognitive decline indicates that pericyte damage may promote the progression of AD.

## Supplementary Information

**Additional file 1: Figure S1.** Study flowchart. **Figure S2.** The potential effects of age, sex and *APOE* genotype on CSF sPDGFRβ levels.

## Data Availability

The dataset supporting the findings of this study is available from the Neurodegenerative Disorder Research Center upon reasonable request.
