# Supplementary material for: Changes in CSF sPDGFRβ level and their association with blood–brain barrier breakdown in Alzheimer’s disease with or without small cerebrovascular lesions
Source: Alzheimers Res Ther. 2023 Mar 14;15:51. doi: 10.1186/s13195-023-01199-5 (PMC10012584; doi:10.1186/s13195-023-01199-5)
Supplement: Supplementary file 1 — Additional file 1: Figure S1. Study flowchart. Figure S2. The potential effects of age, sex and APOE genotype on CSF sPDGFRβ levels. [file 13195_2023_1199_MOESM1_ESM.pdf]

Figure S1

Study flowchart

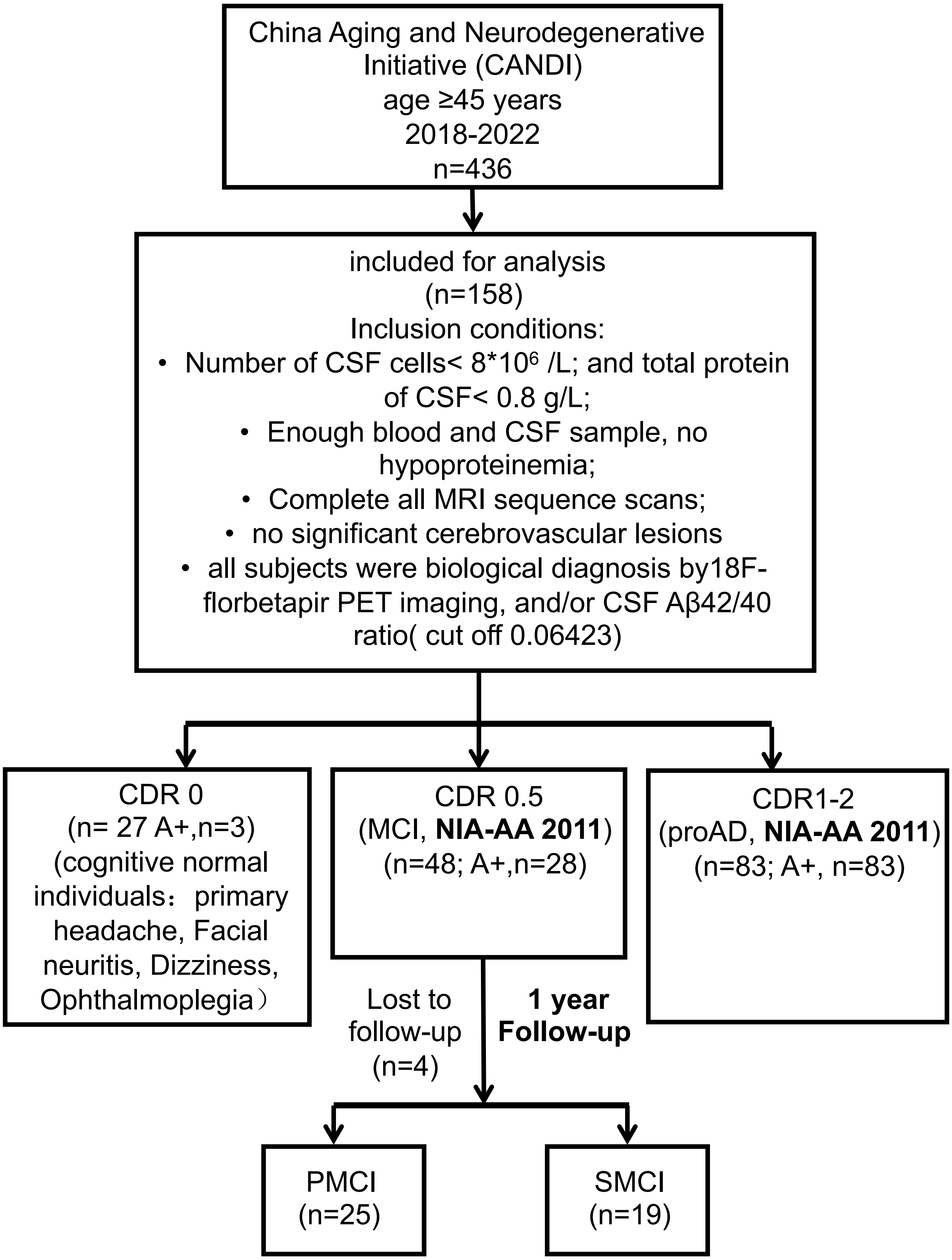

Figure S2

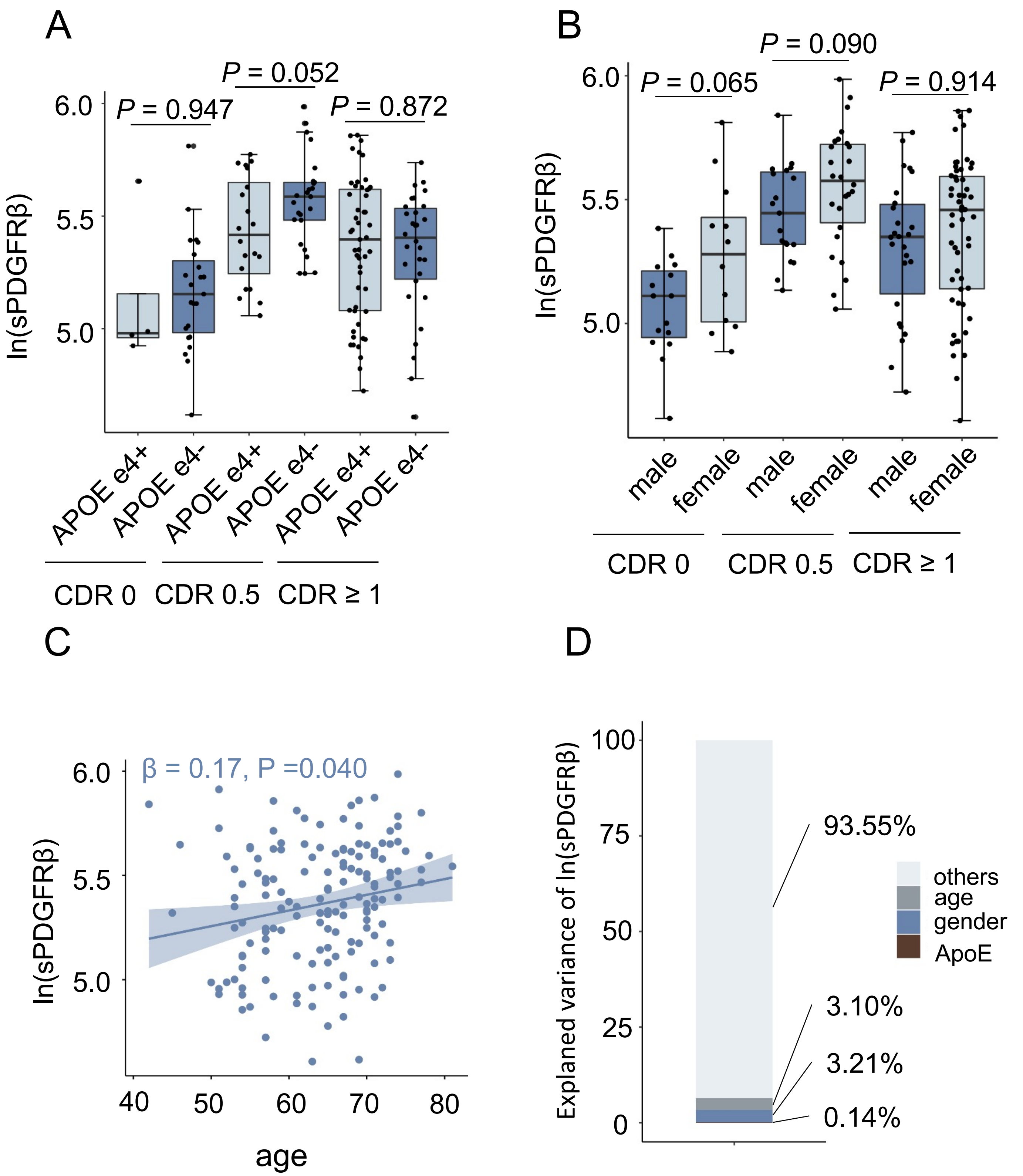

Figure S2. The potential effects of age, sex and *APOE* genotype on CSF sPDGFRβ levels. (A) Comparison of CSF sPDGFRβ levels between *APOE* genotype subgroups in CDR 0, CDR 0.5 and CDR 1-2 group. *P*-values were assessed by a one-way analysis of covariance (ANCOVA) adjusted by age, sex and CSF Aβ42/40. $p < 0.05$ was considered statistically significant. (B) Comparison of CSF sPDGFRβ levels between male and female in CDR 0, CDR 0.5 and CDR 1-2 group. *P*-values were assessed by a one-way analysis of covariance (ANCOVA) adjusted by age, *APOE* genotype and CSF Aβ42/40. $p < 0.05$ was considered statistically significant. (C) *P*-values were assessed by multiple linear regression model, adjusted by sex, *APOE* genotype and CSF Aβ42/40. $p < 0.05$ was considered statistically significant. (D) Contributions of age, sex and *APOE* genotype to the variance of CSF sPDGFRβ levels.
